# What works, why and how? A scoping review and logic model of rural clinical placements for allied health students

**DOI:** 10.1186/s12913-020-05669-6

**Published:** 2020-09-14

**Authors:** Anna Moran, Susan Nancarrow, Catherine Cosgrave, Anna Griffith, Rhiannon Memery

**Affiliations:** 1 Department of Rural Health, 49 Graham St, Shepparton, VIC 3630 Australia; 2 Albury Wodonga Health, Borella Road, Albury, NSW 2640 Australia; 3 Services for Austalian Rural and Remote Allied Health (SARRAH), Unit 4/17 Napier Close, Deakin, ACT 2600 Australia; 4 Southern Cross University, Gold Coast Airport, Terminal Dr, Bilinga, QLD 4225 Australia

**Keywords:** Allied health, Rural, Placement, Work integrated learning, Recruitment, Scoping review, Program logic

## Abstract

**Background:**

Allied health services are core to the improvement in health outcomes for remote and rural residents. Substantial infrastructure has been put into place to facilitate rural work-ready allied health practitioners, yet it is difficult to understand or measure how successful this is and how it is facilitated.

**Methods:**

A scoping review and thematic synthesis of the literature using program logic was undertaken to identify and describe the contexts, mechanisms and outcomes of successful models of rural clinical placements for allied health students. This involved all empirical literature examining models of regional, rural and remote clinical placements for allied health students between 1995 and 2019.

**Results:**

A total of 292 articles were identified; however, after removal of duplicates and article screening, 18 were included in the final synthesis. Australian papers dominated the evidence base (*n* = 11). Drivers for rural allied health clinical placements include: attracting allied health students to the rural workforce; increasing the number of allied health clinical placements available; exposing students to and providing skills in rural and interprofessional practice; and improving access to allied health services in rural areas. Depending on the placement model, a number of key mechanisms were identified that facilitated realisation of these drivers and therefore the success of the model. These included: support for students; engagement, consultation and partnership with key stakeholders and organisations; and regional coordination, infrastructure and support. Placement success was measured in terms of student, rural, community and/or program outcomes. Although the strength and quality of the evidence was found to be low, there is a trend for placements to be more successful when the driver for the placement is specifically reflected in the structure of the placement model and outcomes measured. This was seen most effectively in placement models that were driven by the need to meet rural community needs and upskill students in interprofessional rural practice.

**Conclusion:**

This study identifies the factors that can be manipulated to ensure more successful models of allied health rural clinical placements and provides an evidence based framework for improved planning and evaluation.

## Background

Most health workers live and work in cities, yet almost half of the world’s population currently live in rural and remote areas [1]. Challenges attracting and retaining a full complement of health workers in rural and remote communities is widely recognised as being a significant contributor to rural residents experiencing poorer health outcomes than their metropolitan counterparts [1, 2].

The World Health Organisation (WHO) recommends a number of ways to address the issue of under-supply of health professionals in rural areas, including national policy, regulatory interventions, financial incentives, personal and professional support and the education of health students [2]. This research focuses specifically on Australia’s rural allied health (AH) workforce pipeline, while acknowledging there are also similar and different issues and incentives for medical and nursing workforces.

While there is no universally accepted understanding of what professions are considered part of the AH workforce, it is generally understood as not including the medical, nursing or dental professions. “Allied health professionals are university qualified practitioners with specialised expertise in preventing, diagnosing and treating a range of conditions and illnesses. Allied health practitioners often work within a multidisciplinary health team to provide specialised support for different patient needs” [3] . In Australia, one state has recently recognised 27 allied health professions and has identified them as either therapy focussed professions (such as physiotherapy, occupational therapy, speech pathology, podiatry, dietetics, social work, psychology, exercise physiology and more) or science focussed professions (medical laboratory science, radiology, nuclear medicine, orthoptics, pharmacy, sonography and more) [4].

In support of WHO recommendations, countries like Australia have invested heavily in closing the rural-metropolitan health gap. This has been done through increasing the number of health students and, more specifically, investing in educating students closer to rural communities, bringing students to rural communities, and matching curricula with rural health needs [2].

Significant funding has been directed towards University Departments of Rural Health (UDRHs), of which there are currently 16 located across rural Australia [5]. The UDRH program aims to provide education and training facilities in non-metropolitan centres with the aim of helping attract health professionals to practise in rural and remote communities [6]. In 2013, an estimated 18% of annual university enrolments in 10 leading health disciplines accessed UDRH clinical placements [7]. The capacity for the current education system to accommodate the minimum clinical training or work integrated learning hours required for course accreditation and subsequent professional registration for a (now) large supply of health students, however, is severely limited [8]. In particular, the growth in new courses across AH in Australia means that there is now extreme competition for access to clinical placements in all settings [8].

Rural clinical placements (RCPs) or rural work integrated learning opportunities [in this article the term RCPs will be used] for AH students provide a number of benefits, including: a clinical placement opportunity to meet course accreditation and professional registration requirements; developing skills for (rural) practice; influencing consideration of rural employment; and providing unique learning opportunities [9–12]. Therefore, RCPs offer a potential solution to the placement difficulties for universities whilst simultaneously helping to address broader issues of rural health workforce supply and rural readiness to practise [12]. Often RCPs for health students attempt to achieve all these goals, thus creating a complex set of circumstances where it is difficult to understand or measure how success is facilitated.

There is, however, a paucity of well-synthesised evidence that reflects the complexity of the RCP environment for AH students, in particular why and how particular models of RCP work [2] and how these models impact student, service, patient and key workforce outcomes [13]. It has been argued that traditional systematic reviews, which impose a strict hierarchy of evidence, rarely reflect the complexity of the context in which the interventions are operationalised [14–16]. As such, there is a growing argument that a fuller synthesis of ‘relationships, mechanisms and meaning’ within the evidence base is required by managers and policy makers [17].

## Methods

### Aim, design and setting of the study

Given the complexity of factors that can influence the development, implementation and outcomes of RCPs [12, 18–26], this research is underpinned by a logic framework [27] to identify and illustrate how different elements of RCPs and associations between elements may impact on the outcomes and ultimately the ‘success’ of RCPs.

A preliminary search of PROSPERO, the Cochrane Database of Systematic Reviews and the JBI Database of Systematic Reviews and Implementation Reports was conducted, and no current or in progress systematic reviews on the topic were identified. The objectives of this research are to identify different models of RCP for AH students; to better understand the drivers, contexts, mechanisms and outcomes of these models; and how these elements come together and interact to influence the ‘success’ of the RCP. The end goal of this research is to provide universities, UDRHs and placement sites with clarity around the elements of RCPs they could strengthen according to the outcome they wish to influence. The findings are synthesised using a logic model to identify a guiding framework that can be applied across a range of contexts for the development of sustainable, quality RCPs.

The scoping review was conducted in accordance with the Joanna Briggs Institute methodology for systematic scoping reviews [28] using the Preferred Reporting Items for Systematic reviews and Meta-Analyses extension for Scoping Reviews (PRISMA-ScR) Checklist.

### Review questions

Using a realist perspective [27], the overarching review question is: what AH RCP models currently exist for AH students and what AH RCP models are more successful?

To answer this question, the following sub-questions were developed:

- What are the key drivers of (or needs underpinning) regional, rural and remote clinical training placements for AH students?
- In what types of contexts do AH RCPs take place (e.g. setting, staffing, organisation, structure)?
- What mechanisms (barriers and facilitators) are required for successful AH RCPs?
- What success measures have been used to capture the impact or effectiveness of AH RCPs?
- What is the relationship between drivers, contexts, mechanisms and outcomes?

### Participants

The review considered studies that included AH students and those on interprofessional (IP) placements with other non-AH disciplines (such as medicine or nursing). The search did not include clinical placement studies concerning only nursing or only medical students. A broad definition of allied health was used, with professional titles taken from Services for Australian Rural and Remote Allied Health (SARRAH), the AH portfolio of the New South Wales Health Education Training Institute, the Victorian Department of Health and Human Services, AH workforce and the Allied Health Professions Australia (AHPA) websites (see Table 1 for all terms used).

**Table 1:** Search strategy^a^

Process	Detail
Sampling strategy	Selective: samples databases from medicine, nursing, allied health and social science fields within specified limits.
Type of study	All, quantitative research (randomised controlled trial, controlled clinical trial, controlled before and after study, uncontrolled before and after study), qualitative (grounded theory, ethnography, action research, exploratory approaches, phenomenology, and systematic reviews).
Approaches	Subject searching, citation searching, contact with authors.
Range of years	January 1995–May 2019.
Limits	English, human.
Inclusion and exclusions^b^	Inclusion: empirical study of an intervention aimed at allied health^c^ student clinical placements undertaken in regional, remote and/or rural areas. Exclusions: developing country health care, non-empirical research (grey literature, commentary, editorial, discussion piece), conference abstracts, not allied health (medicine, nursing, dental), not rural/remote/regional, not clinical placement interventions or models, not theses/dissertations.
Terms used^c^	‘Clinical fieldwork’ OR ‘workplace learning’ OR ‘Student Placement’ OR ‘Work practicum’ OR ‘Clinical placement’ OR ‘Field work’ **AND** ‘Audiologists’ OR ‘Art therapists’ OR ‘Chiropractors’ OR ‘Dietetic Technicians, Registered’ OR ‘Dietitians’ OR ‘Electroneurodiagnostic Technologists’ OR ‘Exercise Physiologists’ OR ‘Emergency Medical Technicians’ OR ‘Diabetes Educators’ OR ‘Lactation Consultants’ OR ‘Childbirth Educators’ OR ‘Phlebotomists’ OR ‘Medical Technologists’ OR ‘Medical Laboratory Technicians’ OR ‘Music Therapists’ OR ‘Cytotechnologists’ OR ‘Laboratory Personnel’ OR ‘Occupational Therapists’ OR ‘Occupational Therapy Assistants’ OR ‘Ophthalmic Technologists’ OR ‘Optometrist’ OR ‘Orthopedic Technologists’ OR ‘Orthoptists’ OR ‘Prosthetists’ OR ‘Osteopaths’ OR ‘Pharmacist’ OR ‘Pharmacy Technicians’ OR ‘Physical Therapist Assistants’ OR ‘Physical Therapists’ OR ‘Physician Assistants’ OR ‘Physiotherapists’ OR ‘Podiatrists’ OR ‘Psychologists’ OR ‘Ultrasound Technologists’ OR ‘Radiologic Technologists’ OR ‘Radiation Therapy Technologists’ OR ‘Radiology Personnel’ OR ‘Radiographers’ OR ‘Nutritionists’ OR ‘Nuclear Medicine Technicians’ OR ‘Recreational Therapists’ OR ‘Surgical Technologists’ OR ‘Speech-Language Pathologists’ OR ‘Speech-Language Pathology Assistants’ OR ‘Social Workers’ OR ‘Respiratory Therapists’ OR ‘Registered Care Technologists’ OR ‘Health Educators’ OR ‘Dialysis Technicians’ OR ‘Allied Health Personnel’ OR ‘Allied Health Professional’ **AND** ‘Remote’ OR ‘Regional’ OR ‘Rural’
Electronic sources	Academic Search Premier; CINAHL; EMCARE; InfoRMIT:Health Collection; MEDLINE; ProQuest.

^a^Adapted from STARLITE principles for reporting systematic literature reviews [29]; ^b^detailed in Fig. 2 decision tree; ^c^Allied Health terms taken from SARRAH, Allied Health portfolio of HETI, Allied Health Professions Australia (AHPA) websites (www.sarrah.org.au; http://www.heti.nsw.gov.au/programs/allied-health/allied-health-professions-in-nsw-health/ and www.ahpa.com.au)

### Concept

The review considered studies that explored models of clinical placement. It did not consider studies that tracked longitudinal rural practice intentions of AH practitioners (AHPs) as these studies cannot be attributed to a single particular rural placement model.

### Context

The review considered studies that were undertaken in regional, rural or remote contexts in Australia and in other developed countries, as defined by the United Nations’ World Economic Situation and Prospects country classification [30]. It did not consider studies from metropolitan or urban contexts or from developing countries.

### Types of sources

The review considers both experimental and quasi-experimental study designs, observational studies, qualitative studies and systematic reviews but not theses, dissertations or grey literature. Studies published in English since 1995 were included. Table 1 describes the search strategy and inclusion criteria in full.

### Search strategy

The search strategy targeted published studies. An initial limited search of MEDLINE and CINAHL was undertaken to identify articles on the topic. The text words contained in the titles and abstracts of relevant articles and the index terms used to describe the articles were used to develop a full search strategy (Table 1). The search strategy, including all identified keywords and index terms, was then adapted for each included information source. Reference lists were not screened for additional studies.

The databases searched included: Academic Search Premier; CINAHL; EMCARE; InfoRMIT:Health Collection; MEDLINE and ProQuest. Other unpublished studies, research reports and grey literature were not used for this review.

### Study selection

**Fig. 1:**
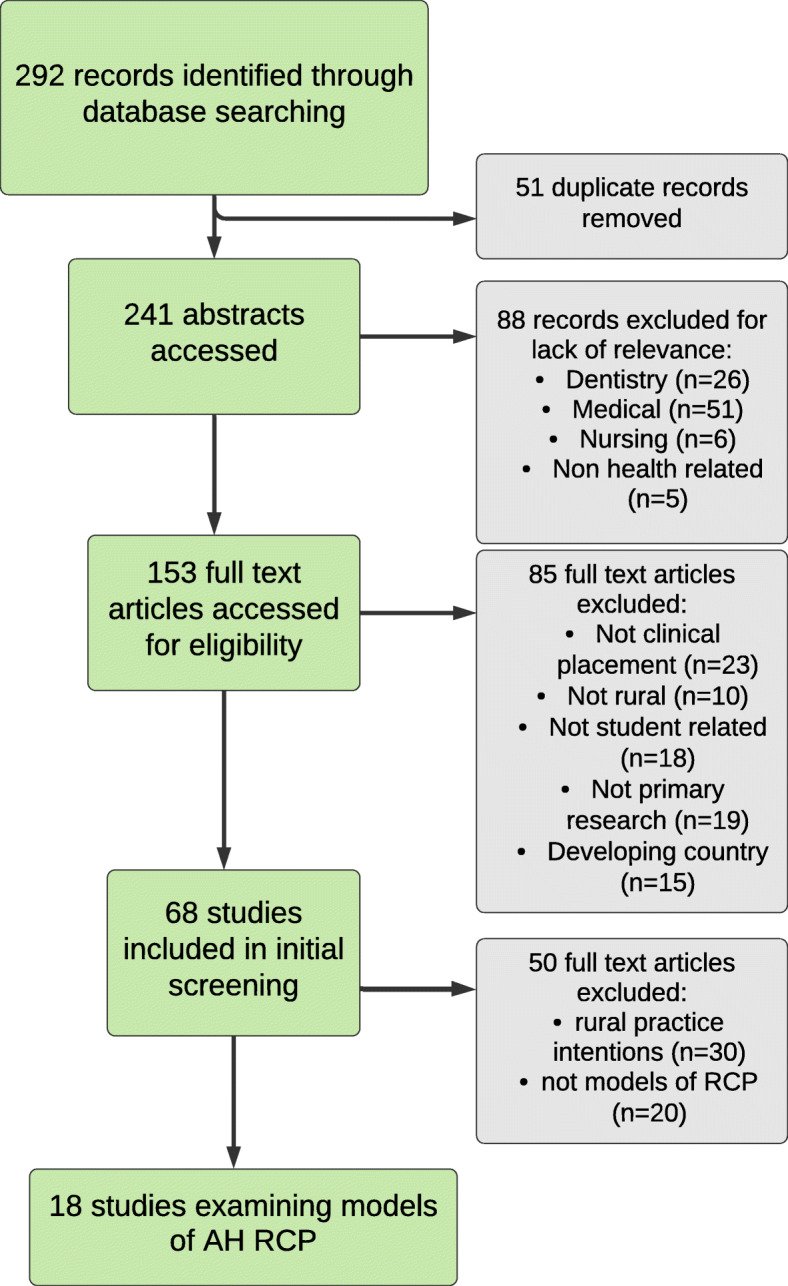
PRISMA diagram

Following the searches, all identified citations were collated and uploaded into EndNote version 7 and duplicates removed. Titles and abstracts were screened by four independent reviewers for assessment against the inclusion criteria for the review (AM, SN, RM, CC; see Table 2). Potentially relevant studies were then retrieved in full and assessed in detail against the inclusion criteria by four independent reviewers (AM, CC, SN, RM). Reasons for exclusion of full text studies that did not meet the inclusion criteria were recorded on the preferred reporting items for systematic reviews and meta-analyses (PRISMA) statement [31]. The results of the search are presented in a PRISMA flow diagram (see Fig. 1).

**Table 2:** Abstract screening process

Process	Decision		
1. Does the paper examine a model(s) of clinical placement?	Yes – Go to 2	No – Exclude	Cannot Tell – Exclude
2. Does the study examine regional, rural and/or remote areas in a developed country^a^?	Yes – Go to 3	No – Exclude	Cannot Tell – Get full paper
3. Does the paper relate to the allied health professions?	Yes – Go to 4	No – Consider for Background	Cannot Tell – Get full paper
4. Does the paper describe an empirical research study or evaluation (including systematic reviews)?	Yes – Include Paper	No – Consider for Background	Cannot Tell – Exclude
5. Does the study provide detail of the model of clinical placement?	Yes – Include paper	No – Consider for Background	Cannot tell – get full text

^a^According to the United Nation’s World Economic Situation and Prospects (WESP) country classification for 2019 [30]

### Data extraction and synthesis

Data were extracted by four independent reviewers (AM, SN, RM, CC) using a logic framework. The data extracted included specific details about the population, concept, context, study methods and key findings relevant to the review objective using pre-defined program logic headings: ‘drivers’ (or needs underpinning), ‘context’ (setting, staffing, organisation, structure of RCPs), ‘mechanisms’ (barriers and facilitators) and ‘outcomes. The data extraction strategy was not modified during the process of extracting data from each included study. Papers were appraised using Joanna Briggs Institute (JBI) critical appraisal tools and the mixed methods assessment tool (MMAT) [32]. Data were synthesised using thematic analysis with the final logic model synthesised using colour coded charting of themes across the logic headings (Additional file 1).

### Data presentation

Extracted data is presented in tabular form with narrative used to describe findings using program logic headings (Additional file 1).

## Results

In total, 292 articles were identified. After removal of duplicates and article screening, 69 papers were considered for inclusion, with 18 included in the final synthesis (Fig. 1 PRISMA). Australian papers dominated the evidence base (*n* = 11) (Table 3 and Additional file 1). The dataset(s) supporting the conclusions of this article is (are) included within the article (and its additional file(s).

**Table 3:** Characteristics of included studies

No.	Reference		Study characteristics			
	Citation no.	Authors, year, title	Country	Allied Health Group	Study Design	Structure & organisation of RCP
**Placements designed to expose students to rural practice**						
1	[33]	Brown, Macdonald- Wicks, Squires, Crowley & Harris (2015) *An innovative dietetic student placement model in rural New South Wales, Australia*	Australia	Dietetics	Cross sectional: audit of 10 years of student placement survey data	Students undertake year-long attachment to rural area - living in one town while completing coursework, research project and placements in region
2	[34]	Capstick S, Beresford R, Gray A. (2008) *Rural pharmacy in New Zealand: effects of a compulsory externship on student perspectives and implications for workforce shortage.*	New Zealand	Pharmacy	Uncontrolled Before-After: A single group of pharmacy students was surveyed, pre- and post-externship, with subjective, self-reported, non-matched responses being recorded	Single-site program where students observed and participated in all activities of the pharmacy practice
3^a^	[35]	Page & Hamilton (2015) *Pharmacy students’ perceptions of a non-traditional rural placement: A pilot programme*	Australia	Pharmacy	Quasi-qualitative: The students’ daily reflections and detailed postplacement reflection were analysed using a qualitative thematic methodology.	2-week observational placement in rural community working with a range of disciplines. Weekly meeting with rural pharmacy academic. Final year students.
4	[36]	Paterson, McColl & Paterson (2004) *Preparing allied health students for fieldwork in smaller communities.*	Canada	Occupational therapy & Physiotherapy	Uncontrolled Before-After: Evaluation of pre and post placement questionnaires measuring student attitudes toward living and working in smaller communities following a three-tiered intervention: 5-day pre-placement workshop; weekly teleconferenced support; financial assistance.	Single-site program supported by pre-placement workshop for students, teleconferencing during the placement and financial assistance.
5	[37]	Wolfgang, Dutton & Wakely (2014) *Creating positive rural experiences for occupational therapy students*	Australia	Occupational Therapy	Quasi-qualitative: Occupational therapy student placement feedback was collated from an online University of Newcastle Department of Rural Health (UoNDRH) student survey they are asked to complete.	Four sites programs - occupational therapy student placed at a single site - supported by UDRH provided training & support and opportunities to participate in community development project
**Placements designed to address community needs**						
6	[38]	Allan, O’Meara, Pope, Higgs & Kent (2011) *The role of context in establishing university clinics*	Australia	Multiple allied health professions	Qualitative (consultative inquiry): literature & document review; site visits & interviews with key stakeholders.	University clinics include: on-campus university clinic provided by a single professional group; Outreach services offered to another site, coordinated through university clinic; partnership with local health agencies.
7	[39]	Averett, Carawan & Burroughs (2012) *Getting “tillerized”: traits and outcomes of students in a rural community field placement*	United States of America	Social Work	Qualitative: process evaluation study using interviews and focus groups	A ‘macro’ rural placement for social work students in an underprivileged rural area with no on-site field instructor and minimal structure. Macro and micro experiences requiring number of professional social work roles.
8	[40]	Boucaut (1998) *Health education activities conducted by physiotherapy students on field trips to rural areas: a case study*	Australia	Physiotherapy	Descriptive case study / reflective opinion piece using student and academic reflections against the Ottawa Charter of health promotion.	Students planned, implemented and evaluated a program of health promotion for a rural community
9	[41]	Frakes K-A, Brownie S, Davies L, Thomas JB, Miller M-E, Tyack Z. (2014) *Capricornia Allied Health Partnership (CAHP): a case study of an innovative model of care addressing chronic disease through a regional student-assisted clinic*	Australia	Multiple AHPs	Cross sectional (plus descriptive): routine data capture of key outcomes over a 12-month period	Student- run clinic, based on Wagner’s chronic care model, where students work in an interprofessional clinical environment to deliver outpatient ‘chronic disease early intervention and management’ services under supervision
10	[42]	Frakes K-A, Brownie S, Davies L, Thomas J, Miller M-E, Tyack Z. (2014) *Experiences from an interprofessional student-assisted chronic disease clinic.*	Australia	Multiple AHPs	Qualitative: Structured interviews were undertaken between students and a clinical educator (other than their primary supervisor) on the last day of their clinical placement	Student- run clinic where students work in an interprofessional clinical environment to deliver outpatient ‘chronic disease early intervention and management’ services under supervision. 2–10-week placement.
11	[18]	Jones D, Grant-Thomson D, Bourne E, Clark P, Beck H, Lyle D (2011) *Model for rural and remote speech pathology student placements: Using non-traditional sites and partnerships*	Australia	Speech Pathology	Cross Sectional: Longitudinal routine data capture of referrals to, consumer access to and use of student service: Each consultation was documented on a standard form, reviewed by the speech pathologist and filed in school records.	Student-run clinics in rural primary schools and aged care/disability services. Students work in pairs running clinics supervised by local therapists.
12	[43]	Kirby S, Held FP, Jones D, Lyle D. (2018) *Growing health partnerships in rural and remote communities: what drives the joint efforts of primary schools and universities in maintaining service-learning partnerships?*	Australia	Speech Pathology	Mixed methods: parallel convergent mixed methods design that combined data analysis from qualitative interviews and online quantitative social network surveys (unvalidated). Participants included speech pathology academics from source universities; host site academics; host site school principals and teaching staff; local site and state education officials; clinical speech pathologists who were engaged as supervisors.	Service-learning placement: students provide classroom based paediatric communication impairment service with supervision from external health service and support from university.
13	[44]	Moosa & Schurr (2011) *Reflections on a Northern Ontario Placement Initiative*	Canada	Speech Language Pathology	Descriptive case study / reflective opinion piece	Under the guidance of the clinical supervisors, the SLP students developed the services and resources requested by the communities, and the programming materials to be shared with the school and hospital staff
**Placements designed to provide students with a specific skill set (Interprofessional)**						
14	[45]	Cragg B, Hirsh M, Jelley W, Barnes P. (2010) *An interprofessional rural clinical placement pilot project*	Canada	Multiple AHPs, nursing & medicine – (physiotherapy, and spiritual care).	Mixed Methods. All students, preceptors, and facilitators participated in semi-structured interviews, and the Interdisciplinary Education Perception Scale (IEPS; Luecht, Madsen, Taugher, & Petterson, 1990), that measures interprofessional attitudes, was administered to students and preceptors pre and post placement	Usual clinical placement supplemented with weekly, one-hour IP education sessions guided by two local facilitators. The sessions were case-based and structured using elements of collaborative learning for students.
15	[46]	Guion WK, Mishoe SC, Taft AA, Campbell CA. (2006) *Connecting allied health students to rural communities*	United States of America	Multiple AHPs - physician assistant, health information management, occupational therapy, physical therapy, and respiratory therapy	Mixed methods project evaluation. Most of the data are based on responses to open-ended questions from student participants, program administrators, and clinical site supervisors.	Rural IP clinical rotation where IP teams of students explored health care access and availability problems.
16	[47]	Gum LF, Richards JN, Walters L, Forgan J, Lopriore M, Nobes C, et al. (2013) *Immersing undergraduates into an interprofessional longitudinal rural placement*	Australia	Multiple AHPs: Nutrition and Dietetic, Speech Pathology and Paramedics	Qualitative: exploration of student perspectives of rural Interprofessional placements through focus groups and self-reflection.	; Placement supplemented with Interprofessional participation in a joint fortnightly Interprofessional learning practicum. Types of activities in the Interprofessional program included case studies, role plays, journal club, work shadowing and invited speakers.
17	[48]	McNair R, Stone N, Sims J, Curtis C. (2005) *Australian evidence for interprofessional education contributing to effective teamwork preparation and interest in rural practice.*	Australia	Multi-professional - AHPs, nursing and medicine (physiotherapy, pharmacy)	Uncontrolled Before - After: before after measurement of student learning outcomes using Barr’s educational outcomes framework for the Interprofessional setting.	Students worked in small Interprofessional teams of 2–4 in rural community health settings supplemented with Joint home visits, observation of team working. Online discussion forum and worked on a joint project.
18	[49]	Mu K, Chao CC, Jensen GM, Royeen CB. (2004) *Effects of interprofessional rural training on students’ perceptions of interprofessional health care services.*	United States of America	Multi-professional - Occupational Therapy, Physiotherapy, Pharmacy and Paraprofessionals (OT assistants and PT assistants)	Mixed methods: Quasi-experimental design using before after measurement of student learning outcomes (IEPS scores), self-assessment tool AND qualitative data collected using a reflection journal and debriefing notes.	Short- & long-term programs involving Interprofessional teams spending time as a team in various activities e.g. community visits, shad-owing activities with clinicians, volunteer activities.
3^a^	[35]	Page AT, Hamilton SJ. (2015) *Pharmacy students’ perceptions of a non-traditional rural placement: A pilot programme*	Australia	Pharmacy	Quasi-qualitative: The students’ daily reflections and detailed postplacement reflection were analysed using a qualitative thematic methodology.	2-week observational placement in rural community working with a range of disciplines. Weekly meeting with rural pharmacy academic. Final year students.

^a^Included in both interprofessional and exposure to rural practice placement models

### What are the key drivers of rural clinical placements for allied health students?

#### Macro – policy level drivers

The evidence base identified macro (policy) level drivers aimed at increasing the size of Australia’s rural allied health workforce to address issues relating to rural health inequality and underservicing of rural areas [33, 34, 36, 37, 41, 43, 45, 47, 49]. As such, the primary macro level driver for RCP identified in the literature concerned the need for attracting AH students to rural health employment upon graduation. A smaller driver was identified which related to the capping of university places and increasing the number of student placement opportunities or placement capacity [37].

#### Meso – university level drivers

A key driver within the university sector for innovation in RCPs and increasing access to more RCP opportunities is the capacity to provide sufficient placement opportunities for its students [38, 40, 46, 49, 50]. Jones et al. stated ‘*There are few placement opportunities nationally across the UDRH network for allied health disciplines such as speech pathology.’* [50], p52. Equally, the literature demonstrates that within the university sector, the provision of RCPs is driven by a commitment to increasing the supply for the rural AH workforce [33–37, 40, 44] and, related to this, ensuring graduates are work-ready for rural employment [38, 43]. For example Wolfgang et al. state *‘Creating positive rural experiences for occupational therapy students on placement could potentially improve the recruitment and retention of occupational therapists in rural and remote areas … and influence occupational therapy students’ decisions to work rurally.’* [37], p204.

Universities were also driven by a commitment to improving access to AH services in rural areas through student clinics or student provision of services whilst on placement [38–44, 50]. Allan et al. for example describe how university clinics were proposed as one way to increase the number of clinical placements available for AH students, while simultaneously providing healthcare to rural communities [38]. . Further, the university sector is also driven to supply RCPs as a unique learning opportunity for students where they can learn particular skills that are key to student competency, such as interprofessional practice [35, 45–49] or cultural competence in working with minority and/or vulnerable population groups. Gum et al. assert that ‘Rural communities provide an ideal context for student exposure to interprofessional clinical practice and an experience of its importance.’ [47], p2.

#### Micro – student and health service drivers

There was only one example in the literature where the driver for the placement was to attract more students to undertake rural placements [39]. The literature did not detail any drivers for the provision of RCP from a clinical educator/supervisor perspective. For students and supervisors, the literature more frequently assessed the *impact* of a rural placement.

These drivers were aligned to three distinct models of RCP identified in the evidence:

1. Placements designed to expose students to rural practice, rural health issues and rural lifestyle, and provide training in rural clinical skills (*n* = 5 [33–37];)
2. Placements designed to address community needs or fill gaps in service provision in rural and remote areas (*n* = 8 [18, 38–44],)
3. Placements designed to provide students with a specific skill set (n = 5 [35, 45–49],)

##### Placements to expose students to rural practice, rural health issues and rural lifestyle, and provide training in rural clinical skills

The key attributes of these models are summarised in Additional file 2. Placements designed to expose students to rural practice, rural clinical skills, rural health issues and rural lifestyle ranged from 1-year voluntary experiences in the fourth year of study [33] to 1-week compulsory placements in the third year of study [34].

##### Placements to meet community needs or fill gaps in service provision in rural and remote areas

Often termed, ‘service learning’ or ‘role emerging’ placements [18, 38–44], the placement for speech pathology students in Broken Hill, Australia is an example of a placement designed to meet community needs [50]. Student-run clinics in primary schools around Broken Hill were developed as a placement option for final year students to address concerns raised by the community about the lack of paediatric speech pathology services in the region [50]. As described in the eight studies examining placements to meet community needs, often these types of placements send groups of students to non-traditional placement sites such as schools or aged care facilities. Supervision is often less intensive, delivered as group supervision and therefore peer learning is frequently utilised to drive learning from placement.

##### Placements that provide students with a specific skill set

The placements providing students with a specific skill set all related to RCPs designed to expose students to IP practice and to improve IP skills among students [35, 45–49]. These placements varied in structure; however, they tended to offer both discipline specific supervision and specific IP opportunities in group situations.

### In what types of contexts do allied health rural clinical placements take place?

AH RCPs take place in a variety of settings and are organised and structured in a number of different ways and are designed to meet some or all of the identified drivers (Additional file 2). In summary, the following contextual elements were identified: the duration of the placement; single or multiple students (or multiple disciplines); practice setting; supervision model; mode of supervision; externally supported/facilitated placement; learning purpose; learning approach; level of choice (compulsory or voluntary); and the year of study in which placement is undertaken. There was little consistency in contextual features within each model of RCP, with no studies providing information on *all* identified features. Placements designed to meet community needs had the most consistent features, with a trend for multiple students to be placed at one time [38, 40–42, 44, 50]. Similarly, placements designed to provide IP skills tended to involve students from multiple disciplines [35, 46–49].

### What mechanisms are responsible for successful delivery of rural allied health clinical placements?

Fifteen different mechanisms relating to the delivery of RCPs (Table 4, Additional file 3) were identified: 1) support for students; 2) support and recognition for supervisors; 3) external funding or sponsor; 4) sustained funding; 5) regional coordination/infrastructure and support (e.g. UDRH); 6) coordination role between university and placement site; 7) stakeholder engagement, consultation and partnership; 8) needs/demand analysis; 9) support for university placement staff; 10) selection criteria/student traits; 11) resourcing; 12) support from registration bodies and/or professional associations; 13) evidence based approach to placement model; 14) regular program planning, evaluation and feedback; and 15) student autonomy.

**Table 4:** Mechanisms for delivery of different placement models

No	Mechanism	Description
1	Support for students	Multiple papers [33, 35–37, 39–44, 46, 47, 49] identified student support as: Information booklets and maps for the locality; travel and/or accommodation costs paid for; daily student allowance provided; induction provided at the beginning of placement; orientation session and tutorials; discussion of learning objectives; discussion of key concepts of rural health practice; accurate communication about what *clinical* experience in rural practice will offer to students; duration; pre placement reflection of personal strengths/weaknesses; post placement debrief opportunity; 1:1 supervision at the end of every clinical session; clusters of students being placed together; for IP placements, understanding of professional identity prior to placement; internet access; phone coverage; access to a library; provision of social opportunities;
2	Support and recognition for supervisors	Provision of supervisor courses for local clinicians; providing support to supervisors during clinical placements; and provision of tutorial programs for students run by the UDRH/Rural Clinical Schools or universities. One paper identifies ongoing difficulties with health staff recruitment and retention impacting on capacity to provide consistent support for student supervision, particularly in rural areas where departments are relatively small [37]. Wolfgang et al. [37] describe how the UDRH provides support and education to clinical supervisors.
3	External funding or sponsor	Guion et al. [46] for example describe receipt of funding from an ‘interdisciplinary grant’ to set up the placement model. Frakes et al. describe the effect of receiving intermittent pockets of state and federal government funding on the ability to maintain their placement model. Kirby et al. (2018) reiterate the importance of ongoing funding for placements designed to address community needs and the need to embed placements into government health and education policy to ensure sustainability
4	Sustained funding	Frakes et al. [41] identify that capacity to implement and evaluate the impact of sustainable RCPs that require collaboration between multiple stakeholders is keenly affected by whether or not funding sources are sustained [41, 42].
5	Regional coordination / infrastructure and support	For example the Australian University Departments of Rural Health (UDRH) function as a single coordination point for the whole region and all the health organisations – ‘a one-stop shop for student placements’ that involves streamlining administrative procedures, maintaining links with service partners, clinical supervisors, feeder universities and students [36, 37, 40, 41].
6	Coordination role between university and placement site	Several papers [36, 37, 41, 47] emphasized the importance of a central broker, advocate or ‘go-between’ in the success of implementing ‘collaborative fieldwork’ models that can increase the capacity of a clinical educator to take multiple students at one time. For example Wolfgang et al. [37] describe how a unit coordinator was responsible for meeting regularly with students or telephoned those in remote locations, coordinated the placement with the university and field work site, provided support and training to supervisors and organised accommodation and transport .
7	Stakeholder engagement, consultation and partnership	The importance of ‘building meaningful partnerships’ and ‘monitoring that all roles and visions are clear and understood’ were essential components of engagement with stakeholders when devising and delivering rural IP clinical placements. Kirby et al. [43] describe trust as a key factor for success: whereby high levels of trust was facilitated by close relationships between stakeholders which in turn was facilitated by social connection in the local community. Kirby states that the combination of work and social connection enriched levels of interaction and facilitated partnerships. The enabled a commitment to be investing and sharing resources. A Memorandum of Understanding to meet unmet need underpinned the partnership.
8	Needs / demand analysis	As identified by Allan’s study [38] describing university clinics, where needs analyses are not conducted there is a risk that the clinic may not provide a sufficient amount or range of clients due to poor geographic positioning of clinic within the campus, sporadic and ineffective marketing and/or lack of range of clients/problem types.
9	Support for university placement staff	Two studies [38, 41] describe the need to adequately support academic staff who run university clinics as their role is often stretched to cover both clinic operations as well as an academic load. Key to successful running of university clinics is also year-round running of the clinic, making support for academic staff even more pertinent to their success.
10	Selection criteria / student traits	Moosa and Shurr [44] describe a placement opportunity for students to develop speech pathology resources in extremely remote and under resourced communities in Canada. They iterated the importance of a selection process to ensure the students had the aptitude and character to cope with the demands of the placement and the ‘hands-off’ supervision model utilised. The authors stated the following requirements for students to undertake the placement: “Interest in rural issues/working rurally, strong academic record, clinical placement evaluations that identified strong professional and ethical conduct, exceptional interpersonal communication skills, rapid integration of feedback, independent problem solving, critical thinking skills” (p.162). Interest in rural practice was also cited as a selection criterion [44].
11	Resourcing	Adequate resourcing for RCPs refers to the infrastructure, time, resources and staffing required to plan, develop, coordinate and deliver the placement such as: providing the placement venue (e.g. school/health service), keeping track of and coordinating all student placements within the health service/community setting and organising and delivering structured education and supervision opportunities (e.g. integrated clinical debrief sessions; group interprofessional sessions; case studies; online activities; and journal clubs). Ongoing resourcing was linked closely to ongoing external funding, which was particularly important for placements designed to address community needs [34].
12	Support from registration bodies and/or professional associations	One author cites that Interprofessional competencies need to be part of placement requirement/university requirement as expressed by one participant “clinical training requirements are set by the universities who set requirements for placements—they don’t require cross discipline work, so the hospital won’t provide it” [43].
13	Evidence based approach to placement model	Frakes et al. [41, 42] describe using reviews of evidence and inviting international experts to present evidence and collaborate in research evaluating placement effectiveness in an effort to maintain quality placements.
14	Regular program planning, evaluation and feedback	Regular evaluation against needs assessment is key to sustainability and success of placement, in particular for placements designed to meet unmet community need. Drawing from implementation science literature, Frakes et al. for example describe the need for a focus on evaluating all aspects of a new model (context, processes and interactions and capacity to sustain). The Capricornica model therefore uses of multi-level evaluation and feedback loops as mechanisms to monitor sustainability and success by collecting impact data around student, staff, patient, referrer and health service outcomes [41, 42]
15	Student autonomy	Student autonomy over determining community needs (conducting needs analyses) or developing the services and resources requested by the communities was key to student learning outcomes, particularly for placements designed to meet community need [40, 44].

The most commonly reported mechanisms across all placements were support for students [33, 35, 39–43, 46–49] and stakeholder engagement, consultation, and partnership [33, 38, 40, 42, 43, 45–47, 50]. The least common were student autonomy [40, 44], sustained funding [41, 43], and support for university placement staff [38, 43]. These mechanisms have been mapped against the three different models of placement and are described below (Table 4 and Additional file 3).

#### Placements designed to increase student exposure to rural practice

The most common mechanisms reported for placements designed around exposure to rural practice were support for students [33, 35–37], regional coordination/infrastructure and support (e.g. presence of a UDRH) [33, 35, 37], and support and recognition for supervisors [33, 37]. Support for students included provision of information booklets and maps for the locality; travel and/or accommodation costs paid for; daily student allowance provided; induction provided at the beginning of placement; orientation session and tutorials and more (Table 4).

#### Placements designed to address community needs

Community focused placements had a much stronger emphasis on community needs. Therefore, a needs/demand analysis [38, 40, 41, 50] and stakeholder engagement, consultation, and partnership (in particular, development of ‘community – academic partnerships’) [18, 38, 40, 42–44] were key to delivering these placements. For example, engagement with the local community in Broken Hill, Australia identified a need for paediatric speech pathology to help improve educational outcomes in children [50]. Sustained funding was also identified as a key mechanism for success and sustainability for the Capricornica chronic care placement model but was rarely realised [41].

#### Placements designed to provide students with a specific skill set

Placements designed around IP skill acquisition described the combined need for both support for students and supervisors [47, 48], stakeholder consultation [45–47], and the designation of a coordinator role that liaises with both the university, placement site and other stakeholders [35, 47, 48]. IP acquisition placements are reported to be particularly resource intense. As such, further key mechanisms for successfully delivering these placements include the availability of funding and support from a funded agency (e.g. rural clinical school) to ensure adequate resourcing for planning, implementation and supporting students [35, 47].

### What measures have been used to capture the impact or effectiveness of different models of clinical placements, and what is the strength and quality of this evidence?

The ‘success’ of different placement models was measured in a variety of ways (Table 5 and Additional file 1) and included measurement of: educational and learning outcomes; student outcomes (such as satisfaction with organisation of the placement, the accommodation, information and support provided, and overall enjoyment); rural outcomes (such as intention to work in a rural area, employment in a rural area post study, knowledge and understanding of the rural context, attitude to living and working in a rural area); program outcomes (e.g. satisfaction with accommodation, support, pre-post placement expectations); supervisor outcomes; service and community outcomes (e.g. reduced waiting lists for patients); and/or placement outcomes (e.g. number of placements provided).

**Table 5:** Outcomes evaluated & evidence quality

No.	Reference, Model and Study		Outcomes evaluated										Evidence Quality
	Citation no.		Education / Learning outcomes	Student outcomes	Rural outcomes: Intention to work in rural area	Rural Outcomes: Employ-ment in rural area post study	Rural Outcomes: Knowledge of rural context	Rural Outcomes: Attitude to living and working in rural area	Program outcomes	Supervisor outcomes	Service & community outcomes	Placement outcomes	
**Placements designed to expose students to rural practice**													
1	[33]	Brown, Macdonald- Wicks, Squires, Crowley & Harris (2015)		✓	✓	✓			✓			✓	Moderate
2	[34]	Capstick, Beresford & Gray (2008)			✓		✓	✓	✓				Moderate
3^a^	[35]	Page & Hamilton (2015)	✓	✓			✓					✓	Low
4	[36]	Paterson, McColl & Paterson (2004)		✓				✓					Low
5	[37]	Wolfgang, Dutton & Wakely (2014)	✓	✓			✓						Low
**Placements designed to address community needs**													
6	[38]	Allan, O’Meara, Pope, Higgs & Kent (2011)	✓	✓							✓	✓	Moderate
7	[39]	Averett, Carawan & Burroughs (2012)	✓	✓					✓				Moderate
8	[40]	Boucaut (1998)	✓	✓			✓	✓			✓		Low
9	[41]	Frakes et al. (2014)	✓	✓					✓		✓	✓	Low
10	[42]	Frakes et al. (2014)	✓	✓									Moderate
11	[18]	Jones et al. (2011)	✓	✓			✓				✓	✓	Low
12	[43]	Kirby, Held, Jones & Lyle (2018)							✓		✓		High
13	[44]	Moosa & Schurr (2011)	✓	✓			✓		✓		✓		Low
**Placements designed to provide students with a specific skill set (interprofessional)**													
14	[45]	Cragg, Hirsh, Jelley & Barnes (2010)	✓	✓				✓					Low
15	[46]	Guion, Mishoe, Taft & Campbell (2006)	✓	✓	✓		✓		✓	✓			Low
16	[47]	Gum et al. (2013)	✓				✓						High
17	[48]	McNair, Stone, Sims & Curtis (2005)	✓	✓				✓					Low
18	[49]	Mu, Chao, Jensen & Royeen (2004)	✓				✓						Moderate
3^a^	[35]	Page & Hamilton (2015)	✓	✓									Moderate

^a^Included in both interprofessional and exposure to rural practice placement models

#### Placements designed to expose students to rural practice

These papers commonly reported a variety of student and/or rural outcomes. The evidence from these studies was generally of low quality with mixed, inconsistent results (Table 5 and Additional file 1). One good quality qualitative paper described students’ improved understanding of the rural health context, improved professional skills and greater understanding of the role of other health professionals post rural placement [35], and another uncontrolled before-after paper reported significantly greater interest in rural work post rural placement for both rural and metropolitan origin students [34]. Brown et al. [33] were the only authors to describe the impact of a 12-month immersion experience for dietetic students on employment in rural settings post placement, with 50–100% of graduated students working in rural areas. The quality of this paper however was low.

#### Placements designed to address community needs

Community needs placements were measured in a variety of ways with an equal emphasis on exploring student and learning outcomes and the impact the placement had on the community. The evidence from all of these studies, although generally poor in quality, suggests these types of placements can have a positive impact on addressing community needs [38–44, 50]. For example, the student speech pathology clinic established as part of the Broken Hill, Australia UDRH recorded that 231 primary school aged children were assessed in 2010; 58% of kindergarten children received a speech pathology intervention; and the number of new referrals on the speech pathology service waiting list decreased from 250 clients in September 2009 to eight in September 2010 .

#### Placements that provide students with a specific skill set (interprofessional)

A variety of measures were employed to gauge the success of IP placements with IP educational and learning outcomes featuring consistently [35, 45–49]. IP outcome measures included: student and supervisor perceptions of IP learning outcomes (including Kirkpatrick’s educational outcomes framework) [35, 45, 47, 49]; and the IP education scale measuring student IP attitudes pre and post placement [45, 48, 49]. Most studies described successful IP outcomes for students as a result of their IP placement model situated in a rural area [50].

One study that used the IP education perception scale pre and post placement reported participation in an IP program in a rural community improved student IP scores; increased their understanding of others’ roles; influenced attitudes towards IP practice for students and supervisors; and there was a significant increase in participants’ positive perceptions regarding IP practice after they participated in the project [49]. As identified by McNair et al. [48], the context of the IP placement, described as the intensity of the ‘immersion’ experience, with students having to negotiate an unfamiliar environment to work and live together, also had a significant influence on students’ learning outcomes. Uniquely, Gum et al. [47] identified that rural IPE placements also have a significant influence on student IP interactions with the rural community.

### Strength and quality of the evidence

The majority of studies used a mix of post placement unvalidated self-report questionnaires, student placement activity measures and/or through interviews and focus groups with a variety of participants. Given the variation in outcomes measured and generally poor quality of the evidence (Additional file 1), robust conclusions cannot be drawn regarding the impact of different models of clinical placement, the exception being some of the IP placement studies that utilised validated IP outcome measurement tools [49] and good quality qualitative research designs [47] to explore the impact of the placement on educational IP outcomes.

## Discussion

To overcome known limitations in drawing conclusions from a weak evidence base with significant variation in outcome measurement, this research aimed to capture the complexity of the context in which AH RCPs are operationalised by presenting the evidence using a logic model framework.

The logic model is presented in Table 6 and addresses the final question of the review: What is the relationship between drivers, contexts, mechanisms and outcomes? The logic model highlights the key ingredients that the evidence has identified as desirable for devising, implementing and evaluating a ‘successful’ AH RCP.

**Table 6:** Components of an Allied Health RCP logic model

***Drivers***	***Contexts***	***Mechanisms***	***(Desired) Outcomes***
Attracting students to the rural workforce	Duration (short-term, medium term, block)	Support for students (e.g. accommodation, travel, living expenses)	Intention to work in a rural area (students)
Increasing the number of clinical placements available for AH students	Single or multiple students (or multiple disciplines)	Support and recognition for supervisors	Increased skills and clinical confidence (e.g. rural generalism, interprofessional skills)
Exposing students to and providing skills in rural practice	Mode of supervision (remote or on site)	Sustained funding	Community and service outcomes: reduced waiting lists/increased service capacity
To attract more students to undertake rural placements	Year of study in which placement is undertaken	Regional coordination/ infrastructure and support (e.g. UDRH)	Increased knowledge and understanding of rural issues/context (students)
Increase service capacity in underserved areas/ address community need	Compulsory or voluntary RCP	Coordination/ facilitation roles that mediate/ broker relationships between feeder universities and placement sites	Employment in a rural area post-graduation
Provision of a specific skill set (e.g. interprofessional competence)	Learning approach (e.g. vertical integration, peer supported learning)	Engagement, consultation and partnership with key stakeholders and organisations	Attitude to living and working in a rural area
	Drivers (e.g. driven by local needs or demands of placement site)	Needs/demand analysis prior to establishing the placement	Enhanced interdisciplinary team working (in specific types of placements)
	Practice setting (e.g. community, hospital, public, private, rural, remote, regional)	Academic support for clinical placement staff/ clinical educators on site	Increased supervisor capability
	Learning purpose	Selection criteria/ student traits	Increased placement capacity
	Externally supported/ facilitated placement (e.g. UDRH)	Provision of resourcing and infrastructure	
	Joint/individual supervision (single or multiple supervisors)	Support from registration bodies and/or professional bodies/associations	
		Evidence based approach	
		Regular program evaluation and feedback	
		Student autonomy	

**Fig. 2:**
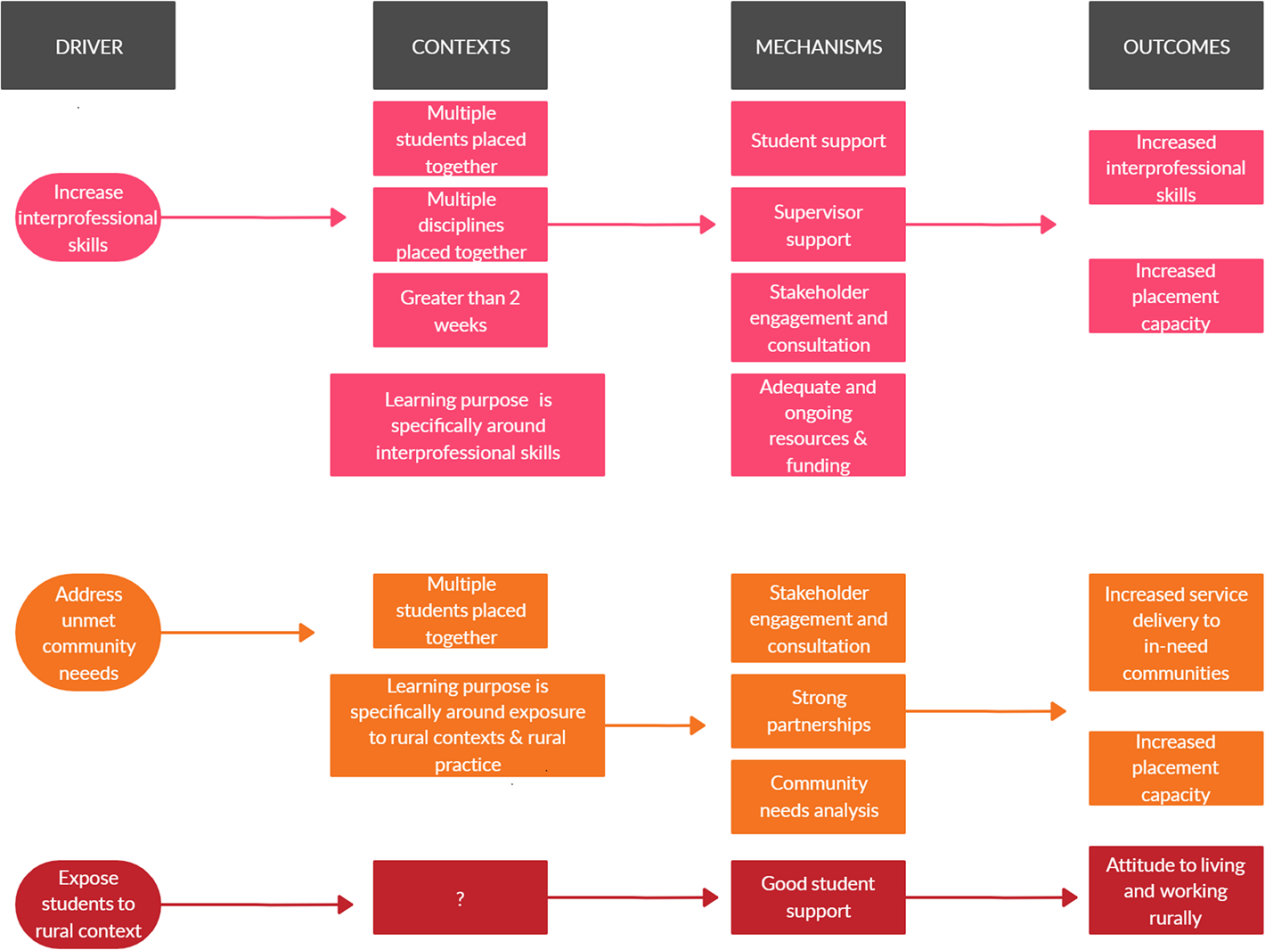
Key relationships in logic model

When connecting drivers with contexts and mechanisms to outcomes (Fig. 2 and Additional file 1), there were only two clear identifiable patterns from the evidence. The first is when the driver for the RCP is to provide students with particular skills and competencies, IP placements undertaken in rural environments have demonstrated improvements in IP competence and increases in the number of student placement opportunities [35, 45–49] (Additional file 1). Key mechanisms that contribute to these outcomes include: the combined need for student and supervisor support, stakeholder consultation and engagement, and provision of adequate and ongoing resources and funding. The contexts or RCP features that support these mechanisms include: multiple disciplines and multiple students being placed on the RCP; an RCP that is 2 weeks or greater; and the RCP has a learning purpose specific to interprofessional skills. The strength of this relationship is supported by moderately good quality research evidence (Additional file 1).

The second identifiable pattern is when the driver for the RCP is to respond to community priorities of unmet health needs, such placements have demonstrated increased service delivery to ‘in-need’ communities and increased placement capacity [38–44, 50]. Key mechanisms that contribute to these outcomes include: development of ‘community–academic partnerships’ alongside a community needs analysis. The contexts or RCP features that support these mechanisms include multiple students being placed on the RCP and the RCP having a learning purpose specific to exposure to rural contexts and rural practice (Additional file 1). The strength of this relationship is supported by moderately good to low quality research evidence (Additional file 1). Whilst not strongly represented in the evidence reviewed, these placements can be costly to provide and sustain [42, 50] and, where communities are very underdeveloped and poorly resourced, student-led interventions were often not realistic or achievable [44]. Therefore, appropriate resourcing should be considered a key mechanism.

These identified patterns are complimented by other rural health workforce research. For example, eight factors have been identified that facilitate the development of effective and sustainable community-academic partnerships. These are (1) creation and nurturing of trust (2) respect for a community’s knowledge (3) community defined and prioritised needs and goals (4) mutual division of roles and responsibilities (5) continuous flexibility, compromise and feedback (6) strengthening of community capacity (7) joint and equitable allocation of resources, and (8) sustainability and community ownership [51]. More recently, the following features have been identified as supporting successful and sustainable community health partnerships in rural and remote Australian locations: 1) identifying and responding to community need, 2) providing services of value, 3) community leadership and innovation, 4) reputation and trust, 5) consistency, and 6) knowledge sharing and program adaptation [25]. These elements should be considered in the development of any RCP concerned with meeting community needs.

In cases when the primary driver for the RCP is to encourage students to work in a rural setting, there was no particular model or, indeed, any consistent contextual components that could be directly linked to increased rates of intention to work in a rural area. While many of the included papers listed attracting students to work in rural settings as being a key driver, only three papers directly measured it [33, 34, 37]. In these papers, there was no consistency of placement features (context) that could be linked to a stated intention to work in a rural area (Additional file 1). Where exposure to rural practice was an identified driver for RCP, this was measured in terms of attitude to living and working in a rural area and/or rural work readiness [33–37]. In these papers, providing good student support was identified as being necessary for ‘successful’ exposure to rural practice (Fig. 2, and see for example Paterson et al. [33] in Additional file 1). This association may lend some weight to the evidence that positive student placement experiences can play a key role in influencing the rate of rural employment of newly graduated nursing and AH practitioners [52]. Noting however that the strength of evidence to support these patterns is limited by the generally low quality of the research (Additional file 1).

There is also growing evidence that immersive RCPs (with multiple students placed for longer placement periods) can influence intention to work rurally [9]. However, other research indicates the decision of health professionals to work in a rural location is not determined simply by background or participation in ‘excellent’ rural placements, but varies between individuals as a result of the complex interaction of many factors [9, 53]. For example one longitudinal Australian study shows that intention to work rurally increases over time, since graduation [54].

We have identified a number of drivers, contextual elements, mechanisms and outcomes, but there are significant gaps. The absence of some of these descriptors from our analysis may be because the articles simply did not include this information or did not see the need to include this information. There is opportunity, therefore, to use the context descriptors identified in this research to inform future reporting of RCP evidence.

### Study limitations

This review has focused on placement models and interventions for rural and remote allied health practitioners and, as such, the mechanisms identified are limited to this group. Future research could consider a realistic evaluation approach that would integrate research examining rural recruitment strategies that include a greater range of health care practitioners and also successful rural recruitment strategies for workers outside the health care industry. The review focused on published, peer reviewed evidence and did not include evaluations or grey literature. Further, articles were excluded where no abstract was available to review. More extensive searching of the evidence base and grey literature may offer greater contextual richness to the logic model described here and allow for development and testing of propositions arising between the contexts, mechanisms and outcomes identified here [55]. The majority of papers identified in this review evaluate novel RCP models. Thus, there is an inherent bias in the conclusions that can be drawn from this review as there are few papers that describe more simple, ‘bread and butter’ RCPs, such as placements offered to just one student, hospital only placements, discipline specific placements or education outcome placements (e.g. paediatric placement in a rural setting). The terms “service learning” and “work integrated learning” which have been more frequently used in recent years in the Australian context to describe clinical placements, were not used in the search strategy. Whilst this review identified a number of studies that used these phrases, the findings of this study may be limited by this omission. Finally, the overall quality of evidence is low, limiting the impact of the study’s findings. Whilst data were extracted and synthesised using program logic to overcome the lack of quality, there remains a critical need to invest in producing high quality research in rural contexts [7, 56].

Future research should focus on testing the logic components identified in this review and developing robust proposition statements that can inform improved decision making around the contexts and mechanisms that contribute towards successful AH RCPs.

## Conclusions

Whilst this review found some evidence to support the proposition that undertaking an RCP may lead to increased intention to practice in a rural area, there is little evidence regarding the type or model or elements of a RCP that can be applied to achieve this. Better quality research of AH RCP models is required. There is a need for more systematic and psychometrically robust measurements of the impact of different models of RCP. There is also a need to utilise more uniform, standardised and validated tools to measure key outcomes of RCPs, such as intention to practice in a rural location, rural work readiness, attitudes towards rural practice, and placement quality. Furthermore, defining, monitoring and consistently measuring sustainability as an outcome of RCPs is required. Finally, improvement in describing placements in a more systematic way to support comparison is necessary. The logic model presented in this paper provides such descriptors.

## Supplementary information

**Additional file 1.** Extracted data and logic model relationships. Extracted data from the literature, evidence review and relationships between data categories.

**Additional file 2.** Context features. Context data from the literature.

**Additional file 3.** Mechanisms. Mechanism data from the literature.

## Data Availability

All data generated or analysed during this study are included in this published article and its supplementary information files.

## Abbreviations

AH: Allied health
AHP: Allied health practitioner
AHPA: Allied Health Professions Australia
HETI: Health Education Training Institute
IP: Interprofessional
JBI: Joanna Briggs Institute
MMAT: Mixed Methods Assessment Tool
PRISMA: Preferred Reporting Items for Systematic Reviews and Meta-analyses
PROSPERO: International Prospective Register of Systematic Reviews
RCP: Rural clinical placement
SARRAH: Services for Australian Rural and Remote Allied Health
UDRH: University Department of Rural Health
WHO: World Health Organisation
